# Iron(II) and copper(II) paratungstates B: a single-crystal X-ray diffraction study

**DOI:** 10.1107/S2053229618010021

**Published:** 2018-10-16

**Authors:** Nadiia I. Gumerova, Anatolie Dobrov, Alexander Roller, Annette Rompel

**Affiliations:** a Universität Wien, Fakultät für Chemie, Institut für Biophysikalische Chemie, Althanstrasse 14, Wien 1090, Austria; b Universität Wien, Fakultät für Chemie, Zentrum für Röntgenstrukturanalyse, Währinger Strasse 42, Wien 1090, Austria

**Keywords:** polyoxometalate, POM, isopolytungstate, three-dimensional structure, crystal structure, dodeca­tungstate, IPOT

## Abstract

Paratungstate B is a common isopolytungstate (IPOT) built of the [W_12_O_40_(OH)_2_]^10−^ anion and exhibits a cluster-like construction of 12 W-centred distorted octa­hedra. Due to a high surface charge density, the paratungstate anion acts as a multidentate ligand forming high-dimensional extended structures, which exhibit unique catalytic and magnetic properties. Two new paradodeca­tungstate B compounds decorated by iron(II) or cop­per(II), namely Na_5_Fe_2.5_[W_12_O_40_(OH)_2_]·36H_2_O (**Na_5_Fe_2.5_paraB**) and Na_4_Cu_3_[W_12_O_40_(OH)_2_]·28H_2_O (**Na_4_Cu_3_paraB**), have been synthesized by a convenient aqueous solution method, and structurally characterized by single-crystal and powder X-ray diffraction, IR spectroscopy, elemental analysis and thermogravimetric analysis. Both compounds crystallize in the triclinic *P*  space group. In both compounds, the [W_12_O_40_(OH)_2_]^10−^ polyanion acts as a multidentate ligand that links transition-metal and sodium cations, forming a three-dimensional framework.

## Abstract 2

Two new isopolytungstates, Na_5_Fe_2.5_[W_12_O_40_(OH)_2_]·36H_2_O and Na_4_Cu_3_[W_12_O_40_(OH)_2_]·28H_2_O, have been prepared and structurally characterized. The compounds exhibit a three-dimensional structure, in which the paratungstate anions coordinate to Fe^II^ or Cu^II^ ions in a polydentate mode.

## Introduction   

The structural diversity of polyoxometalates (Pope, 1983 ▸) and their proven applications in catalysis (Wang & Yang, 2015 ▸), nanotechnology (Yamase & Pope, 2002 ▸), electrochemistry (Sadakane & Steckhan, 1998 ▸), materials science (Proust *et al.*, 2008 ▸), mol­ecular magnetism (Clemente-Juan *et al.*, 2012 ▸), macromolecular crystallography (Bijelic & Rompel, 2015 ▸, 2017 ▸; Molitor *et al.*, 2017 ▸) and medicine (Fu *et al.*, 2015 ▸; Bijelic *et al.*, 2018*a* ▸,*b* ▸) have encouraged the synthesis of novel polyanions with promising properties. One of the most common isopolytungstates (IPOTs) is paratungstate B, built of the [W_12_O_40_(OH)_2_]^10−^ anion that is stable in aqueous acidic solution and exhibits a cluster-like construction of 12 W-cen­tred distorted octa­hedra (Evans & Rollins, 1976 ▸; Pope, 1983 ▸). Due to a high surface charge density, the paratungstate anion acts as a multidentate ligand, which can coordinate alkaline (Peresypkina *et al.*, 2014 ▸) and transition-metal cations (Radio *et al.*, 2010 ▸, 2011 ▸; Gumerova *et al.*, 2015 ▸), and also act as a precursor (Sokolov *et al.*, 2012 ▸). By coordinating transition-metal cations, paratungstates can form high-dimensional extended structures, which exhibit unique catalytic (He *et al.*, 2008 ▸; Chen *et al.*, 2017 ▸) and magnetic properties (Li *et al.*, 2008 ▸, 2009 ▸). So far, three paratungstates B with Fe^II^ and nine with Cu^II^ as counter-cations have been successfully synthesized and characterized by X-ray diffraction (Table 1 ▸). We present herein two novel paratungstates B, one with Fe^II^ and one with Cu^II^, namely the double sodium–iron(II) paratungstate Na_5_Fe_2.5_[W_12_O_40_(OH)_2_]·36H_2_O (denoted **Na_5_Fe_2.5_paraB**) and the double sodium–copper(II) paratungstate Na_4_Cu_3_[W_12_O_40_(OH)_2_]·28H_2_O (denoted **Na_4_Cu_3_paraB**), which were synthesized by a convenient aqueous solution method.

## Experimental   

### Synthesis and crystallization   

The reagents were used as purchased from Sigma–Aldrich without further purification.

#### Synthesis of **Na_5_Fe_2.5_paraB**   

Iron powder (0.112 g, 2 mmol) was added to a solution (15 ml) of Na_2_WO_4_·2H_2_O (3.96 g, 12 mmol), which was acidified to pH = 2.5 with HCl (1 *M*). The mixture was stirred in an ultrasonic bath, giving a deep-blue solution, which was left to stand closed at room temperature. The pale-red–brown crystals which grew on the beaker walls were collected after three weeks (yield ∼2 g, ∼53%, based on W). Elemental analysis found (calculated) for Fe_2.5_H_74_Na_5_O_78_W_12_ (%): Na 3.13 (3.03), Fe 3.71 (3.69), W 56.8 (58.32).

#### Synthesis of **Na_4_Cu_3_paraB**   

Sodium orthotungstate Na_2_WO_4_·2H_2_O (5.5 g, 16.7 mmol) was dissolved in water (25 ml) and the pH was adjusted to 8 by adding dilute HNO_3_ (1 *M*). An aqueous solution (10 ml) of Cu(NO_3_)_2_·3H_2_O (0.5 g, 2.1 mmol) was then added dropwise, while the pH was maintained between 3.0 and 4.5 with HNO_3_ (1 *M*). The final mixture was filtered (pH = 4.2) and allowed to stand closed at room temperature. Light-blue crystals formed within two months (yield ∼3.5 g, 69% based on W). Elemental analysis found (calculated) for Cu_3_H_58_Na_4_O_70_W_12_ (%): Na 2.62 (2.51), Cu 5.13 (5.20), W 59.1 (60.16).

### IR spectroscopy   

The title compounds were identified by IR measurements on a Bruker Vertex70 IR Spectrometer equipped with a single-reflection diamond-ATR unit (ATR is attenuated total reflectance) in the range 4000–400 cm^−1^.

### TGA measurements   

Thermogravimetric analysis (TGA) was performed on a Mettler SDTA851e Thermogravimetric Analyzer under a nitro­gen flow with a heating rate of 5 K min^−1^ in the region from 298 to 973 K.

### Elemental analysis   

Elemental analysis was conducted using inductive-coupled plasma–mass spectrometry (PerkinElmer Elan 6000 ICP MS) and atomic absorption spectroscopy (PerkinElmer 1100 Flame AAS) in aqueous solutions containing 2% HNO_3_. Standards were prepared from single-element standard solutions of concentration 1000 mg l^−1^ (from Merck, Ultra Scientific and Analytika Prague).

### Powder X-ray diffraction   

Powder X-ray diffraction (PXRD) was performed on a Bruker D8 Advance diffractometer, with Cu *K*α radiation (λ = 1.54056 Å), a Lynxeye silicon strip detector, a SolX energy dispersive detector and a variable slit aperture of 12 mm. The 2θ range was 8–50°.

### Refinement   

In Table 2 ▸, the crystallographic characteristics of the two new paratungstates B and the experimental conditions of the data collection and refinement are reported. The positions of the independent H atoms were obtained by difference Fourier techniques and were refined with free isotropic displacement parameters.

Fixed isotropic displacement parameters for all H atoms with a value equal to 1.5*U*_eq_ of the corresponding O—H group atom were assigned. Restrained distances for *D*—H bonds were applied to avoid short *D*—H⋯H—*D* interactions. To force correct bonds, specified bonds were added to or removed from the connectivity list.

The disordered water molecules in the coordination spheres of atom Na1 in **Na_4_Cu_3_paraB** and of atoms Na4 and Na5 in **Na_5_Fe_2.5_paraB** were refined with two positions with fixed occupancy factors of 0.5.

In **Na_4_Cu_3_paraB**, part of the disordered water molecules were not modelled and the disordered density was considered using the *OLEX2* (Dolomanov *et al.*, 2009 ▸) implementation of *BYPASS* (a.k.a. SQUEEZE; Spek, 2015 ▸). The modelled electron density is consistent with approximately four water molecules per unit cell.

The structures have been deposited with the Inorganic Crystal Structure Database (ICSD) (http://www2.fiz-karlsruhe.de/icsd_home.html) under collection numbers 434558 and 434559.

## Results and discussion   

The syntheses of **Na_5_Fe_2.5_paraB** and **Na_4_Cu_3_paraB** were carried out with W^VI^-to-*M* ^II^ ratios of W:Fe = 12:2 and W:Cu = 12:1.5, and a pH of 2.5 for **Na_5_Fe_2.5_paraB** and 4.2 for **Na_4_Cu_3_paraB**, which are different from previously reported conditions (Table 1 ▸) and made it possible to obtain com­pounds with new Fe–Na and Cu–Na compositions. The presence of Na^I^ as counter-cation in paratungstates B, together with Cu^II^ or Fe^II^, have been observed previously both in excess and in deficiency of the transition-metal ion in the reaction mixture, which had a pH in the range 3.5–6.5 (Table 1 ▸). This allows one to conclude that crystallization of paratungstates B as double-alkali–transition-metal salts is more preferable than crystallization of pure transition-metal paratungstates B, regardless of the starting molar ratios of the components and the pH of the reaction system.

**Figure 1:**
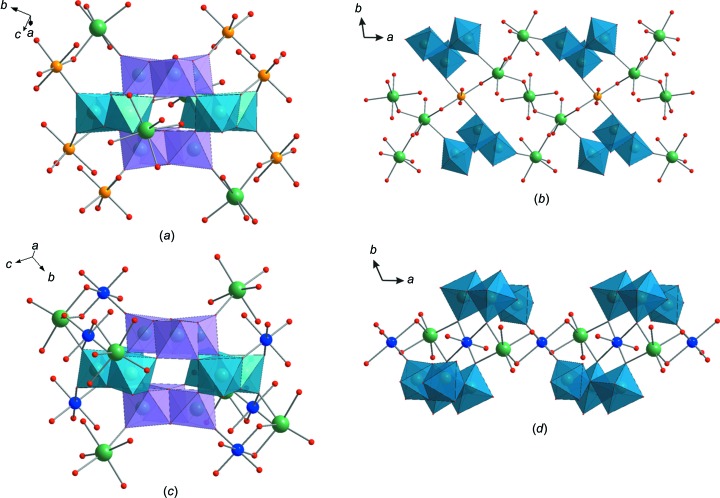
Structural elements in **Na_5_Fe_2.5_paraB** and **Na_4_Cu_3_paraB**. (*a*) The [W_12_O_40_(OH)_2_]^10−^ anion in **Na_5_Fe_2.5_paraB** connected to four Na^+^ and six Fe^2+^ ions *via* terminal O atoms. (*b*) A fragment of the infinite 1D chain in **Na_5_Fe_2.5_paraB** consisting of Na and Fe polyhedra. (*c*) The [W_12_O_40_(OH)_2_]^10−^ anion in **Na_4_Cu_3_paraB** connected to six Na^+^ and six Cu^2+^ ions *via* terminal O atoms. (*d*) A fragment of the infinite 1D chain in **Na_4_Cu_3_paraB** consisting of Na and Cu polyhedra. Colour code: {WO_6_} are light-blue or violet octa­hedra, {W_3_O_14_} are blue octa­hedra and {W_3_O_13_} are violet octa­hedra, and Na atoms are green, Fe orange, Cu blue and O red.

The main structural elements of **Na_5_Fe_2.5_paraB** and **Na_4_Cu_3_paraB** are shown in Fig. 1 ▸. Both compounds consist of paradodeca­tungstate B [W_12_O_40_(OH)_2_]^10−^ polyanions (Evans & Rollins, 1976 ▸; Pope, 1983 ▸), sodium and transition-metal cations, and additional water mol­ecules (Fig. 1 ▸). The paratungstate B units observed in **Na_5_Fe_2.5_paraB** and **Na_4_Cu_3_­paraB** are structurally identical to previously reported units (Table 1 ▸).

In **Na_4_Cu_3_­paraB**, there is one-half unit of the POM, which lies on an inversion centre, in the asymmetric unit. For **Na_5_Fe_2.5_paraB**, there are two independent half-POM units in the asymmetric unit.

The centrosymmetric [W_12_O_40_(OH)_2_]^10−^ anion consists of four corner-sharing groups: two {W_3_O_13_} (violet octa­hedra in Figs. 1 ▸ *a* and 1*c*) and two {W_3_O_14_} (blue octa­hedra in Figs. 1 ▸ *a* and 1*c*) units. Each {W_3_O_13_} fragment is formed by three edge-sharing {WO_6_} octa­hedra with a common O atom, while in the {W_3_O_14_} triads, the three edge-sharing {WO_6_} octa­hedra are linearly connected with no common O atom to the three W atoms. In the {W_3_O_13_} groups, each octa­hedron has one terminal O atom, while in the {W_3_O_14_} units, each octa­hedron has two unshared O atoms (Figs. 1 ▸ *a* and 1*c*). The O atoms connected to the W centres can be classified into three groups. The first group is comprised of terminal O atoms (O_t_), each bonded to one W atom. The second group consists of bridging O atoms (O_db_), each connected to two W atoms. There are two types of O_db_, one bridges two W atoms within the same {W_3_O_13_} or {W_3_O_14_} fragment (O_db1_), while the other bridges two W atoms between the different {W_3_O_13_} and {W_3_O_14_} units (O_db2_). The third group contains triply bridging O atoms, linked by three W atoms. The triply bridging O atoms exclusively from {W_3_O_13_} are labelled O_tb1_, whereas the O atoms bridging three W atoms between {W_3_O_13_} and {W_3_O_14_} units are labelled as O_tb2_.

The exact positions of the two protons in [W_12_O_40_(OH)_2_]^10−^ were located previously on triply bridging O atoms of {W_3_O_13_} by neutron diffraction (Evans & Prince, 1983 ▸). Selected bond lengths and angles are presented in Table 3 ▸. All the W atoms in [W_12_O_40_(OH)_2_]^10−^ exhibit the +VI oxidation state, when applying the bond valence sum (BVS) calculations of Brown & Altermatt (1985 ▸). For **Na_5_Fe_2.5_paraB** and **Na_4_Cu_3_paraB**, we got average values of 6.01 and 6.09, respectively. BVS calculations for Fe and Cu sites show that both ions exhibit the +II oxidation state, with a value of 2.12 for Fe and 2.08 for Cu.

**Figure 2:**
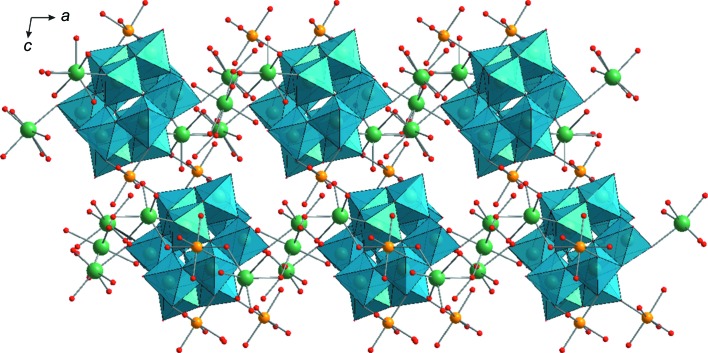
The crystal packing of **Na_5_Fe_2.5_paraB**, viewed along the *b* axis. Colour code: {WO_6_} are light-blue octa­hedra and Na atoms are green, Fe yellow and O red.

In the crystal structure of **Na_5_Fe_2.5_paraB**, the paratungstate anions act as decadentate ligands, which are linked *via* terminal O atoms to six Fe^2+^ and four Na^+^ cations. There are two crystallographically unique iron centres with different coordination modes (Figs. 1 ▸ *a* and 1*b*). The coordination sphere of one type of Fe^2+^ atom (Fe2) is formed by two O_t_ from the belt unit {W_3_O_14_} of one [W_12_O_40_(OH)_2_]^10−^, one O_t_ from the capping {W_3_O_13_} group of a neighbouring polyanion and completed by three H_2_O mol­ecules. The octa­hedrally coordinated second Fe^2+^ atom (Fe1) is linked by two O_t_ from the {W_3_O_13_} units of two neighbouring polyanions, two Na^+^ bridging O atoms and two lattice H_2_O mol­ecules. The Fe1 octa­hedron and three Na(H_2_O)_6_ units from the infinite one-dimensional (1D) chain share a corner, thereby forming a two-dimensional sheet (2D) in the *ab* plane (Fig. 1 ▸ *b*). Neighbouring sheets are connected to each other by Fe2 cations, giving rise to a complicated three-dimensional structure (Figs. 2 ▸ and 3 ▸). It should be noted that the double sodium–iron(II) paratungstate B Na_5_Fe_2.5_[W_12_O_40_(OH)_2_]·36H_2_O reported in this work has the same cationic composition as reported in Na_5_[{Fe(H_2_O)_3_}_2_{Fe(H_2_O)_4_}_0.5_(H_2_W_12_O_42_)]·30H_2_O (Yang *et al.*, 2003 ▸) (Table 1 ▸). However, the minor difference with respect to the water content in these two structures leads to a significant change in the unit-cell parameters (Tables 1 ▸ and 2 ▸) and the motif of crystal packing (Figs. 2 ▸ and 3 ▸).

**Figure 4:**
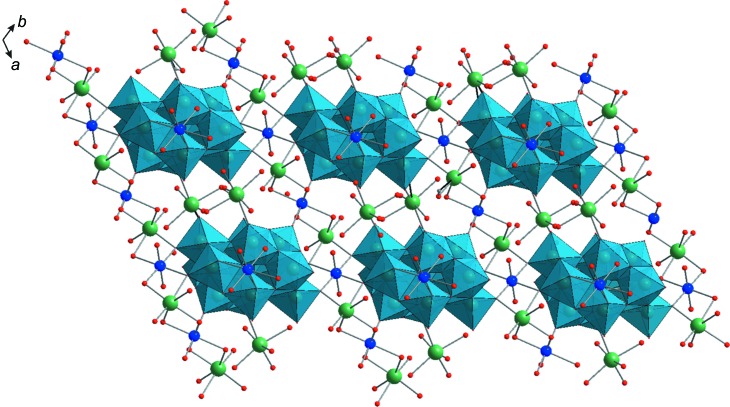
The crystal packing of **Na_4_Cu_3_paraB**, viewed along the *c* axis. Colour code: {WO_6_} are light-blue octa­hedra and Na atoms are green, Cu blue and O red.

**Figure 5:**
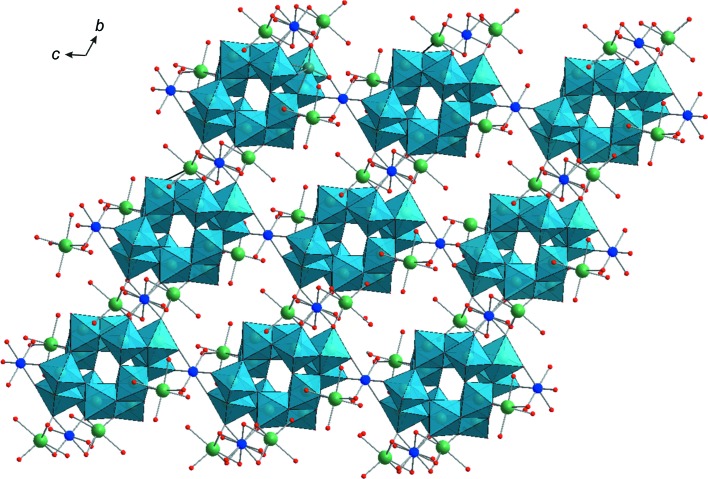
The crystal packing of **Na_4_Cu_3_paraB**, viewed along the *a* axis. Colour code: {WO_6_} are light-blue octa­hedra and Na atoms are green, Cu blue and O red.

In the crystal structure of **Na_4_Cu_3_­paraB**, each paratungstate B anion is coordinated to six Cu^2+^ and six Na^+^ *via* O_t_ and therefore acts as a dodecadentate ligand (Figs. 1 ▸ *c* and 1*d*). There are three crystallographically unique copper centres with different coordination modes. Two (Cu1 and Cu2) out of three Cu^2+^ cations take part in the formation of infinite chains with alternating Na and Cu polyhedra connected by a common edge (Figs. 1 ▸ *d* and 4) and have different coordination environments. The Cu2 atoms are linked by four O_t_ atoms of the belt-fragment {W_3_O_14_} from two neighbouring [W_12_O_40_(OH)_2_]^10−^ anions and two Na^+^ bridging H_2_O mol­ecules. The coordination sphere of Cu3 consists of two O_t_ of the capping {W_3_O_13_} group of a neighbouring polyanion and is completed by four bridging H_2_O mol­ecules. The third Cu1 atom coordinates to four O_t_ atoms of the belt units {W_3_O_14_} from two neighbouring [W_12_O_40_(OH)_2_]^10−^ anions and two H_2_O mol­ecules. The Cu^2+^ ions exhibit a distorted square–bipyramidal coordination geometry with elongated axial distances [2.365 (7)–2.520 (8) Å]. The three-dimensional (3D) structure of **Na_4_Cu_3_-paraB** consists of 2D sheets formed by two chains, namely {[(Na(H_2_O)_2_)_2_W_12_O_40_(OH)_2_]^8−^}_*n*_ and {[Na(H_2_O)_2_–Cu(H_2_O)_2_–Na(H_2_O)_2_–Cu(H_2_O)_4_]^6+^}_*n*_ parallel to the *ab* plane (Fig. 4 ▸). The 2D sheets are connected along the *c* axis by [Cu(H_2_O)_4_]^2+^ cations (Fig. 5 ▸). The two [Na(H_2_O)_5_]^+^ cations, which are connected to O_t_ of one polyanion and do not participate in the formation of sodium–copper chains, are located in 1D tunnels in the structure of **Na_4_Cu_3_-paraB**.

**Figure 6:**
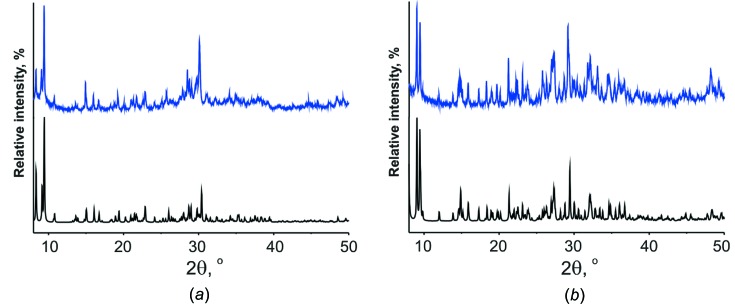
Experimental (blue) and simulated (black) X-ray diffraction patterns of (*a*) **Na_4_Cu_3_paraB** and (*b*) **Na_5_Fe_2.5_paraB**.

The results of the powder XRD patterns of **Na_5_Fe_2.5_paraB** and **Na_4_Cu_3_paraB** have been investigated in the solid state at room temperature (Fig. 6 ▸). The simulated powder diffraction pattern was based on the single-crystal structural data. The simulated peak positions are in good agreement with those observed. A comparison of the experimental and simulated powder diffraction patterns confirms that the POTs structures had been solved accurately and that both products consist of a single phase.

In the IR spectra of **Na_5_Fe_2.5_paraB** and **Na_4_Cu_3_paraB**, the characteristic peaks at 975, 950, 932, 867, 676 and 488 cm^−1^, and at 972, 937, 926, 872, 675 and 493 cm^−1^, respectively, are attributed to the W=O_t_ and W—O—W vibrations in the paratungstate anion, which are in agreement with previously reported data (Table 1 ▸; Qu *et al.*, 2012 ▸). The slight peak displacements are due to the effects of different coordination modes of paratungstate B. The peaks at ∼1600 and 3400 cm^−1^ are attributed to the vibration of water mol­ecules.

**Figure 7:**
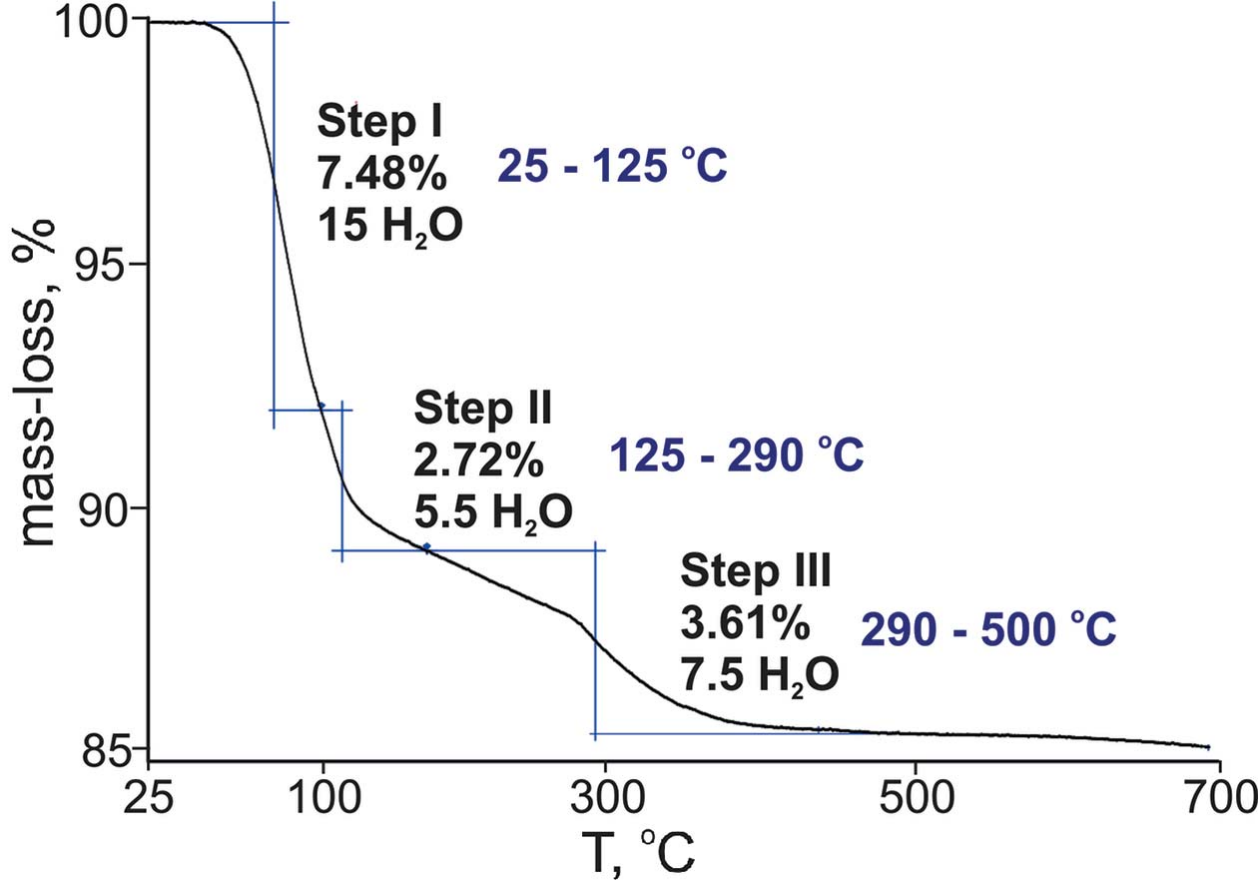
Thermogravimetric curve of **Na_4_Cu_3_paraB**.

The disordered water mol­ecules in **Na_4_Cu_3_paraB** were treated with *SQUEEZE* (Spek, 2015 ▸) and the exact number of water mol­ecules was determined by TGA. The TG curve shows a three-step weight-loss process (Fig. 7 ▸). The first weight loss of 7.48% in the temperature range 25–125 °C corresponds to all lattice H_2_O and water mol­ecules from coordinating Na^+^ and Cu^2+^. The second (2.72%) and third (3.61%) steps in the range 125–500 °C correspond to 13 H_2_O mol­ecules coordinating Na^+^ and Cu^2+^. The total weight loss is 13.83%, which results in the formula Na_4_Cu_3_[W_12_O_40_(OH)_2_]·28H_2_O.

The success in synthesizing **Na_5_Fe_2.5_paraB** and **Na_4_Cu_3_paraB** shows that paratungstate B is a versatile building block, which can be modified by metal sites into high-dimensional architectures and the different connection principle of the transition metals has a big impact on the dimensionalities of the frameworks.

## Supplementary Material

Crystal structure: contains datablock(s) ando209_p-1, mo_ando241_p-1, global. DOI: 10.1107/S2053229618010021/jr3015sup1.cif

Structure factors: contains datablock(s) ando209_p-1. DOI: 10.1107/S2053229618010021/jr3015ando209_p-1sup2.hkl

Structure factors: contains datablock(s) mo_ando241_p-1. DOI: 10.1107/S2053229618010021/jr3015mo_ando241_p-1sup3.hkl

CCDC references: 1843518, 1843519

## Figures and Tables

**Figure 3:**
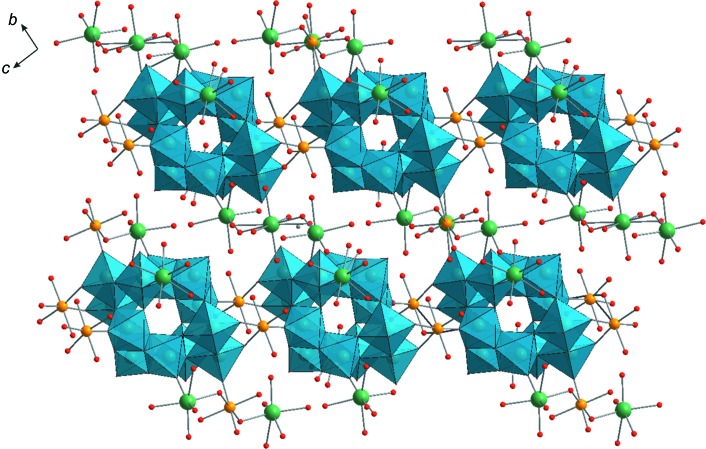
The crystal packing of **Na_5_Fe_2.5_paraB**, viewed along the *a* axis. Colour code: {WO_6_} are light-blue octa­hedra and Na atoms are green, Fe yellow and O red.

**Table 1:** Fe^II^- and Cu^II^-containing paratungstates B [based on the Inorganic Crystal Structure Database (FIZ, Karlsruhe; http://www.fiz-informationsdienste.de/DB/icsd/www-recherche.html) and the Cambridge Structural Database (CSD; Groom *et al.*, 2016 ▸)]

Compounds	Unit-cell parameters *a*, *b* and *c* (Å), and α, β and γ (°)	Volume (Å^3^), *Z* and space group	Synthesis details (source of W; W:*M* ^II^ ratio, with *M* = Fe, Cu; pH)	Reference
**Fe^II^**				
K_6_[{Fe(H_2_O)_4_}_2_(H_2_W_12_O_42_)]·15H_2_O	14.9967 (5), 10.3872 (3), 18.8237 (6); 90, 93.407 (1), 90	2927.1 (2), 2, *P*2_1_/*n*	K_2_WO_4_; 12:1.4; –	Yang *et al.* (2003 ▸)
(H_3_O)_2_[{Fe(H_2_O)_4_Fe(H_2_O)_3_}_2_(H_2_W_12_O_42_)]·20H_2_O	12.1794 (4), 22.4938 (4), 11.6941 (3); 90, 105.731 (2), 90	3083.7 (1), 2, *P*2_1_/*c*	Li_2_WO_4_; 12:1.4; –	Yang *et al.* (2003 ▸)
Na_5_[{Fe(H_2_O)_3_}_2_{Fe(H_2_O)_4_}_0.5_(H_2_W_12_O_42_)]·30H_2_O	12.121 (2), 12.426 (3), 13.247 (3); 68.33 (3), 71.33 (3), 71.44 (3)	1710.7 (6), 1, *P* 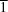	Na_2_WO_4_; 12:1.4; –	Yang *et al.* (2003 ▸)
				
**Cu^II^**				
Na_8_[Cu(H_2_O)_2_(H_2_W_12_O_42_)]·30H_2_O	13.081 (4), 13.160 (6), 20.127 (6); 78.294 (12), 78.524 (11), 72.593 (11)	3201.7 (17), 2, *P* 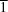	Na_2_WO_4_; 12:2.4; 4.8	Li *et al.* (2008 ▸)
KNa_3_[Cu(H_2_O)_2_{Cu(H_2_O)_3_}_2_(H_2_W_12_O_42_)]·16H_2_O	10.799 (2), 11.914 (2), 13.377 (3); 70.18 (3), 68.07 (3), 64.80 (3)	1410.9 (5), 1, *P* 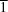	Na_2_[W_12_O_40_(OH)_2_]; 12:2; 3.5	Li *et al.* (2009 ▸)
[{Na_2_(μ-H_2_O)_2_(H_2_O)_6_}{Cu(H_2_O)_2_}{Cu(H_2_O)_4_}_2_{Cu_2_(μ-OH)_2_(H_2_O)_6_}(H_2_W_12_O_42_)]·10H_2_O	10.697 (5), 12.921 (5), 13.653 (5); 73.608 (5), 75.671 (5), 67.748 (5)	1654.4 (12), 1, *P* 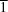	(NH_4_)_6_[W_12_O_40_]; 12:0.4; 6.2	Kong *et al.* (2010 ▸)
[{Na(H_2_O)_4_}_2_{Cu_0.5_(H_2_O)}_4_{Cu_0.5_(H_2_O)_1.5_}_2_(H_4_W_12_O_42_)]·3H_2_O	10.7060 (11), 12.7124 (14), 13.1664 (14); 113.7600 (10), 90.8230 (10), 111.8290 (10)	1493.8 (3), 1, *P* 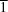	Na_2_WO_4_; 12:3; 6.5	Gao *et al.* (2011 ▸)
[Na_2_(H_2_O)_10_][Cu_4_(H_2_O)_12_(H_2_W_12_O_42_)]·15H_2_O	10.1535 (2), 13.2118 (3), 13.7049 (5); 112.692 (3), 94.771 (3), 102.969 (2)	1623.15 (8), 1, *P* 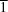	Na_2_WO_4_; 12:36; 4	Qu *et al.* (2012 ▸)
Cu_3_(H_2_O)_8_[H_6_W_12_O_42_]	10.6753 (5), 12.7814 (5), 13.0976 (5); 113.737 (4), 90.433 (3), 112.560 (4)	1482.73 (12), 2, *P* 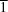	(NH_4_)_6_[W_12_O_40_]; 12:36; –	Chen *et al.* (2017 ▸)
(NH_4_)_8_[Cu(H_2_O)_2_H_2_W_12_O_42_]·10H_2_O	14.278 (5), 15.435 (5), 24.881 (5); 90, 90, 90	5483 (3), 2, *Pbcn*	(NH_4_)_6_[W_12_O_40_]; 12:2.5; 4.8	Zhang (2012 ▸)
Na_2_Cu_3_(CuOH)_2_[W_12_O_40_(OH)_2_]·32H_2_O	10.6836 (4), 12.9066 (6), 13.6475 (5); 73.561 (4), 75.685 (3), 67.666 (4)	1648.68 (12), 1, *P* 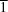	Na_2_WO_4_; 12:7.5; –	Radio *et al.* (2014 ▸)
Na_2_Cu_5_(H_2_O)_24_(OH)_2_[H_2_W_12_O_42_]·10H_2_O	10.7140 (8), 12.9476 (9), 13.6696 (10); 73.56, 75.73, 67.69	1661.8 (2), 1, *P* 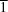	Na_2_WO_4_; 12:20; 3.8	Qu *et al.* (2015) ▸

**Table 2:** Experimental details

	**Na_4_Cu_3_paraBM**	**Na_5_Fe_2.5_paraB**
Crystal data		
Chemical formula	Na_4_Cu_3_[W_12_O_40_(OH)_2_]·28H_2_O	Na_5_Fe_2.5_[W_12_O_40_(OH)_2_]·36H_2_O
*M*_r_	3621.13	3771.27
Crystal system, space group	Triclinic, *P* 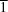	Triclinic, *P* 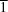
Temperature (K)	100	100
*a*, *b*, *c* (Å)	10.6516 (5), 12.7532 (6), 13.0730 (5)	12.3758 (6), 14.7752 (7), 18.8919 (8)
α, β, γ (°)	113.771 (1), 90.443 (1), 112.502 (1)	92.9341 (14), 100.6938 (14), 94.1698 (15)
*V* (Å^3^)	1473.65 (11)	3378.1 (3)
*Z*	1	2
Radiation type	Mo *K*α	Mo *K*α
μ (mm^−1^)	24.53	21.02
Crystal size (mm)	0.13 × 0.07 × 0.02	0.37 × 0.07 × 0.04

Data collection		
Diffractometer	Bruker APEXII CCD	Bruker D8 Venture
Absorption correction	Multi-scan (*SADABS*; Bruker, 2016 ▸)	Multi-scan (*SADABS*; Bruker, 2016 ▸)
*T*_min_, *T*_max_	0.004, 0.023	0.012, 0.044
No. of measured, independent and observed [*I* > 2σ(*I*)] reflections	11292, 5321, 4720	38068, 12318, 10876
*R*_int_	0.048	0.032
(sin θ/λ)_max_ (Å^−1^)	0.602	0.602

Refinement		
*R*[*F* ^2^ > 2σ(*F* ^2^)], *wR*(*F* ^2^), *S*	0.035, 0.098, 1.05	0.022, 0.058, 1.09
No. of reflections	5321	12318
No. of parameters	463	980
No. of restraints	100	405
H-atom treatment	H atoms treated by a mixture of independent and constrained refinement	H atoms treated by a mixture of independent and constrained refinement
Δρ_max_, Δρ_min_ (e Å^−3^)	2.15, −1.63	1.57, −1.25

Computer programs: *APEX3* (Bruker, 2015 ▸), *SAINT* (Bruker, 2015 ▸), *SHELXS97* (Sheldrick, 2008 ▸), *shelXle* (Hübschle *et al.*, 2011 ▸), *SHELXL2014* (Sheldrick, 2015 ▸), *SHELXL2016* (Sheldrick, 2015 ▸), *OLEX2* (Dolomanov *et al.*, 2009 ▸) and *DIAMOND* (Brandenburg, 2006 ▸).

**Table 3:** Selected bond length and angles (Å, °) in **Na_5_Fe_2.5_paraB** and **Na_4_Cu_3_paraB**

	**Na_5_Fe_2.5_paraB**	**Na_4_Cu_3_paraB**
W=O_t_	1.719 (4)–1.797 (4)	1.710 (8)–1.780 (7)
W—O_db1_	1.888 (4)–2.050 (2)	1.872 (7)–2.103 (7)
W—O_db2_	1.826 (3)–2.166 (3)	1.805 (7)–2.098 (7)
W—O_tb1_	2.201 (3)–2.297 (3)	2.207 (7)–2.273 (7)
W—O_tb2_	1.895 (4)–2.259 (4)	1.882 (8)–2.287 (7)
W⋯W (between corner-sharing WO_6_)	3.649 (4)–3.878 (5)	3.377 (4)–3.688 (2)
W⋯W (between edge-sharing WO_6_)	3.273 (4)–3.352 (4)	3.306 (2)–3.377 (3)
*M* ^II^—O (*M* = Fe or Cu)	2.087 (4)–2.169 (4)	1.918 (7)–2.366 (8)
Na^I^—O	2.302 (11)–2.606 (11)	2.345 (9)–2.519 (13)
O—W—O	70.73 (14)–104.38 (17)	154.94 (15)–177.56 (16)
	70.2 (3)–105.6 (3)	152.8 (3)–178.1 (3)
